# BRCA1 Exon 11, a CERES (Composite Regulatory Element of Splicing) Element Involved in Splice Regulation

**DOI:** 10.3390/ijms150713045

**Published:** 2014-07-23

**Authors:** Claudia Tammaro, Michela Raponi, David I. Wilson, Diana Baralle

**Affiliations:** Human Development and Health, University of Southampton, Southampton SO16 6YD, UK; E-Mails: ct4e10@soton.ac.uk (C.T.); m.raponi@soton.ac.uk (M.P.); d.i.wilson@soton.ac.uk (D.I.W.)

**Keywords:** BRCA1, splicing, unclassified variant, alternative splicing, minigene, exon11

## Abstract

Unclassified variants (UV) of BRCA1 can affect normal pre-mRNA splicing. Here, we investigate the UV c.693G>A, a “silent” change in BRCA1 exon 11, which we have found induces aberrant splicing in patient carriers and *in vitro*. Using a minigene assay, we show that the UV c.693G>A has a strong effect on the splicing isoform ratio of BRCA1. Systematic site-directed mutagenesis of the area surrounding the nucleotide position c.693G>A induced variable changes in the level of exon 11 inclusion/exclusion in the mRNA, pointing to the presence of a complex regulatory element with overlapping enhancer and silencer functions. Accordingly, protein binding analysis in the region detected several splicing regulatory factors involved, including SRSF1, SRSF6 and SRSF9, suggesting that this sequence represents a composite regulatory element of splicing (CERES).

## 1. Introduction

Splicing is the most important step in the expression of genetic information and essential for mRNA to be correctly translated into a protein. During the splicing process, the non-coding sequences (introns) are removed and the coding sequences (exons) are joined together to form the mature mRNA; these reactions take place in the spliceosome [1]. The splicing reaction takes place in two trans-esterifications reactions [2]. These complex mechanisms require the identification of *cis*-acting sequences: the 5' and 3' splice site, the branch point and the polypyrimidine tract, together with additional *cis*-acting elements (situated in the intron or exon) that can act either as enhancers or silencers of splicing. These elements are able to interact with splicing regulatory factors, such as the serine arginine-rich (SR) group and the heterogeneous nuclear ribonucleoproteins (hnRNPs). Exonic splicing enhancers (ESEs) tend to be bound by SR proteins, whereas exonic splicing silencers (ESS) are bound by hnRNP proteins. The interaction of each of these factors allows the inclusion or exclusion of particular exons. Mutations in exonic splicing regulatory regions are found as common events in human pathology [3]. The inclusion or the exclusion of an exon will alter the translated protein, and therefore, alternative splicing may result in aberrant protein isoforms, which can cause disease, including cancer. For instance, studies on *BRCA1* have identified different splicing isoforms associated with breast and ovarian cancer [4,5,6,7,8]. It has also been shown that the relative levels of *BRCA1* isoforms associated with exon 11 alternative splicing differs between normal and cancer tissues [9]. Two key *BRCA1* splicing isoforms have been identified in both breast cancer tissue and cell lines: *BRCA* Δ11 and Δ11q. The Δ11 isoform, generated through skipping of exon 11, is associated with the loss of BRCA1 function, particularly in DNA repair. Exon 11 is required for the binding of RAD51 (the Δ11 isoform is not able to bind RAD51), which alters the capacity to repair DNA double-strand breaks, resulting in the accumulation of cell cycle defects [9,10]. The Δ11q isoform is produced through the use of an internal 5' splice site in exon 11, generating an mRNA containing a partial section of exon 11, with an undefined function, but known to affect cancer cell growth [9]. 

Our work evaluated an unclassified variant (UV) c.693G>A, a synonymous variant in exon 11 of *BRCA1* gene. Synonymous variants create codons that encode for the same amino acid residue, but may affect splicing [11,12]. This variant was identified in a patient with a strong family history of breast cancer and had previously been reported [13] to affect splicing in lymphocytes from another patient [13]. For this purpose, we studied the variant using a previously described *BRCA1* exon 11 minigene for the splicing assay [14]. Our results demonstrate an alteration of the process of splicing, similar to that observed previously by Dias Brandao *et al.* [13]. Site-directed mutagenesis around position c.693 demonstrated a change in the splicing pattern and the presence of a composite regulatory element of splicing (CERES) [15,16] in *BRCA1* exon 11. In particular, *in vitro* binding assays suggest the participation of *SRSF1*, *SRSF6* and *SRSF9* in the regulation of CERES-mediated BRCA1 exon 11 splicing. 

## 2. Results and Discussion

### 2.1. The UV (Unclassified Variant) c.693G>A Affects Splicing of Exon 11

**Figure 1:**
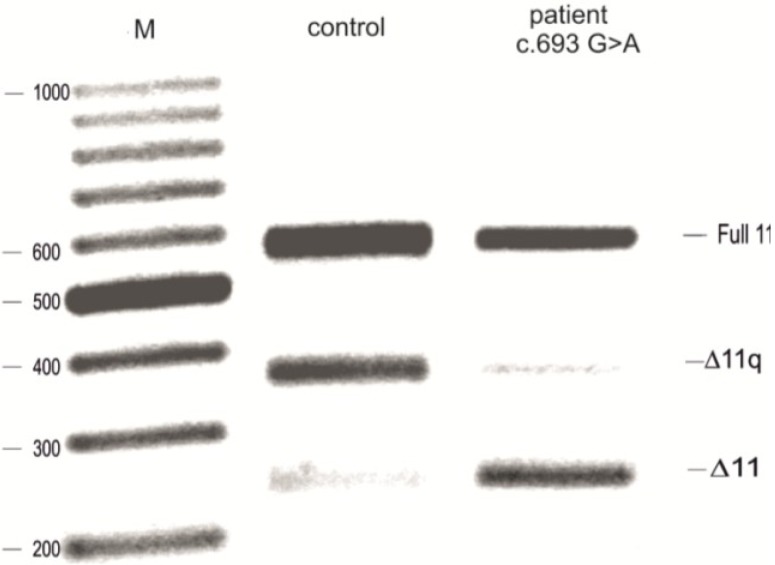
Analysis of BRCA1 cDNA from blood of a patient carrying the variant c.693G>A. Amplification of the cDNA from patient c.693 and the control demonstrated that the variant increases the level of the Δ11 isoform and decreases the Δ11q isoform. M—ladder.

RNA from a patient affected with breast and a strong family history of breast cancer, carrying a synonymous substitution c.693G>A in BRCA1 exon 11, was extracted from a whole blood sample and the splicing products compared with wild-type healthy controls. The splicing products were analysed by reverse transcriptase PCR (RT-PCR) using specific primers to analyse the three splicing isoforms: full 11, Δ11q isoform and the Δ11 isoform (as previously described [14]). As shown in Figure 1, the patient sample, compared to the healthy control sample, shows a relative decrease of full 11 and Δ11q isoform and an increase of Δ11 isoform, suggesting the creation of a new enhancer or the disruption of a silencer interfering with the recognition of the 3' splice site. Based on this theory, a further analysis was performed using a minigene for splicing assay in order to test the effect of the variant in breast cancer cell lines (MCF7).

### 2.2. Minigene Analysis

**Figure 2:**
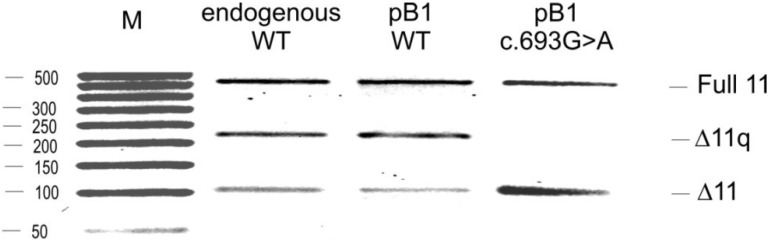
Analysis of BRCA1 minigene. Reverse transcriptase PCR (RT-PCR) (as in Figure 1) from the transfection of endogenous BRCA1 (endogenous wild-type (WT)), the minigene wild-type (pB1WT) and the minigene with the variation (pB1 c.693G>A). To the right of the gel is reported the isoform corresponding to each of the three bands: full-length 11(Full 11), Δ11, Δ11q isoforms. M—ladder.

**Figure 3:**
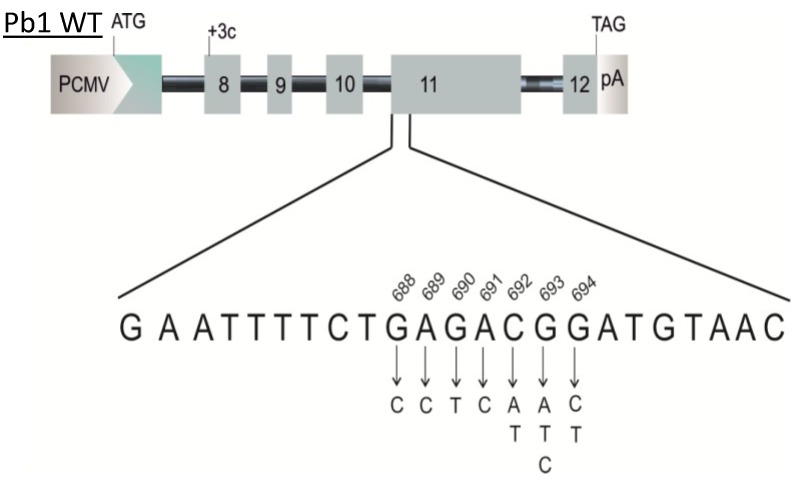
Site-directed representation. Schematic representation of the minigene pB1 WT and the single nucleotide change from nucleotide c.688 to c.694. PCMV, promoter cytomegalovirus; pA = polyA.

**Figure 4:**
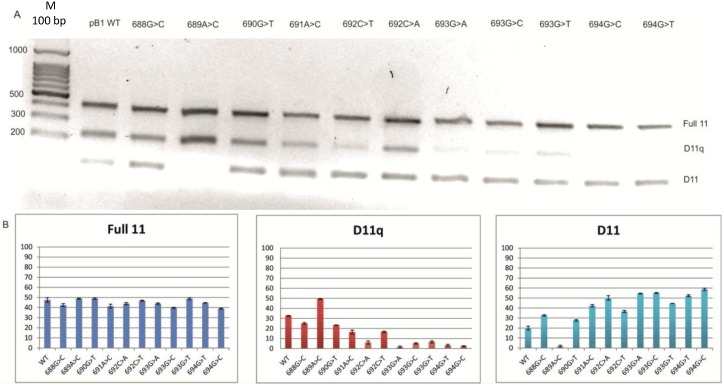
Minigene analysis by site-directed mutagenesis in BRCA1 exon 11. (**A**) The upper diagram shows a 1.5% agarose gel of the single point mutation. Full 11 represents the full 11 isoform, D11q the Δ11q isoform, and D11 corresponds to the Δ11 isoform; (**B**) the lower panel shows the percentage (%) of the full 11, Δ11q and Δ11 isoforms, respectively, calculated against the total expression of the three isoforms. The intensity of the each band was calculated using Image J (National Institute for Health, Bethesda, MD, USA). M—ladder.

**Figure 5:**
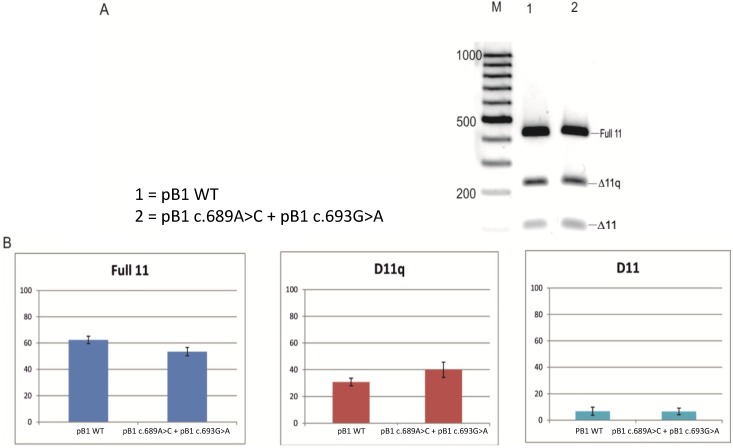
Minigene carrying the double mutant. (**A**) Splicing product for the pB1 WT minigene (**Lane 1**) and the minigene with the double mutant (**Lane 2**). On the right of the gel is a schematic representation of the three splicing isoform corresponding to each of the three bands: the Full 11, the Δ11q and Δ11 isoforms. M is the 100-bp DNA ladder marker; (**B**) The lower panel shows the percentage (%) of the Full 11, Δ11q and Δ11 isoforms, respectively, calculated against the total expression of the three isoforms. The intensity of the each band was calculated using Image J.

To investigate the mechanism by which this mutation is associated with exon 11 skipping, we constructed a minigene with the variant c.693G>A [14]. The pB1 minigene contains the BRCA1 genomic sequence from exon 8 to exon 12 in the pCDNA 3 (+) vector. The minigene with the variant was transfected in MCF7 cells and after 48 h RNA was extracted, retro-transcribed and amplified using specific primers able to discriminate between the three isoforms: full 11, Δ11 and the Δ11q isoform. The splicing products were visualized on 1.5% of agarose gel, and the results are reported in Figure 2. The pB1 wild-type (WT) minigene produces all three splicing isoforms. However, the minigene carrying the variant c.693G>A showed a relative increase of the Δ11 isoform and a decrease of the Δ11q and full 11 isoform, in concordance with the results seen in the patient blood sample (Figure 1). Other cell lines were also tested, and the minigene carrying the variant c.693G>A resembled the change in BRCA1 exon 11 splicing observed in MCF7 cells (data not shown). In order to investigate the regulatory elements, site-directed mutagenesis was performed (Figure 3). Twelve hybrid minigenes were each transfected into MCF7 cell lines and the splicing analysed. All of the variants, except for c.689A>C, showed a proportionate increase in the Δ11 isoform and a decrease in Δ11q compared with the WT minigene (Figure 4). These results provide evidence for a region between nucleotides 688 and 694 that regulates splicing of exon 11. The nucleotide change at position c.689A>C showed a decrease of the Δ11 isoform, suggesting the creation of a new enhancer or the disruption of a silencer. A double mutant was therefore created to determine if the variant c.689A>C could compensate for the synonymous variant c.693G>A. Figure 5 shows that the minigene with the double mutant can rescue the original mutant c.693G>A and recreate the wild-type splicing pattern. 

**Figure 6:**
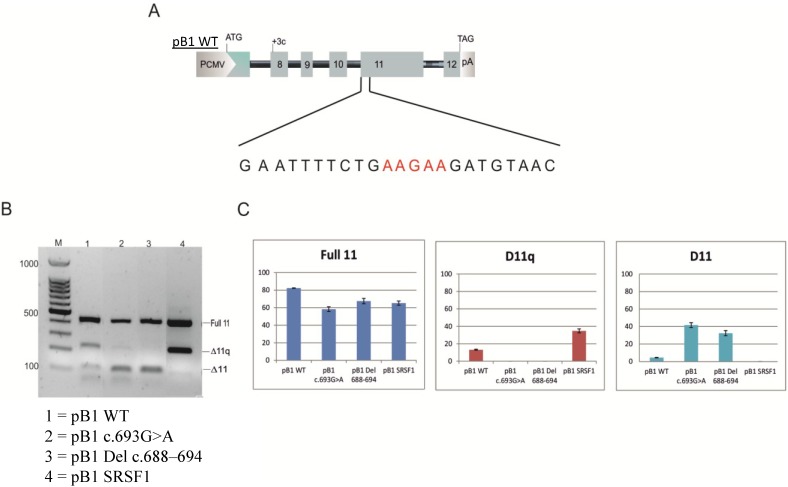
Minigene carrying the deletion c.688–c.694 and the minigene pBSRSF1. (**A**) The figure shows the minigene pB1WT with the sequence AAGAA (represented in red), which has been replaced in the pB1 SRSF1 minigene; (**B**) The splicing product for the pB1WT minigene (**Lane 1**), the minigene carrying the mutation c.693G>A (**Lane 2**), the pB1 minigene with the deletion of the region between c.688 to c.694 (**Lane 3**) and the minigene pB1 SRSF1 (**Lane 4**). On the right of the gel is a schematic representation of the three splicing isoforms corresponding to each of the three bands: the full 11, the Δ11q and the Δ11 isoform. M is the 100-bp DNA ladder marker;and (**C**) The histogram shows the percentage (%) of the Full 11, Δ11q and Δ11 isoforms, respectively, calculated against the total expression of the three isoforms. The intensity of the each band was calculated using Image J.

We also investigated the effects of a deletion of the regulatory region (Δc.688–694) (Figure 6B lane 3). This showed an increase of Δ11 and a decrease of Δ11q and full 11, which is comparable to the minigene splicing of variant c.693G>A, confirming that the region is important for the inclusion of exon 11. 

Bioinformatic analysis using Human Splicing Finder [17,18] strongly suggests that the sequence variant c.693G>A disrupts a binding site for SR proteins (data not shown), in particular SRSF1, SRSF5 and SRSF7, and creates a binding site for hnRNP A1. A minigene was therefore created that included a strong consensus binding site for the common SR protein, SRSF1 (GAAGAAC), between positions c.688 to c.694 (Figure 6A). This was transfected in MCF7 cell lines. Only the full 11 and Δ11q isoforms were spliced, indicating that replacement of a strong binding site for SRSF1 promotes the inclusion of exon 11 (Figure 6B, Lane 4).

### 2.3. Identification of Proteins Binding to the Splicing Regulatory Element of BRCA1 Exon 11

**Figure 7:**
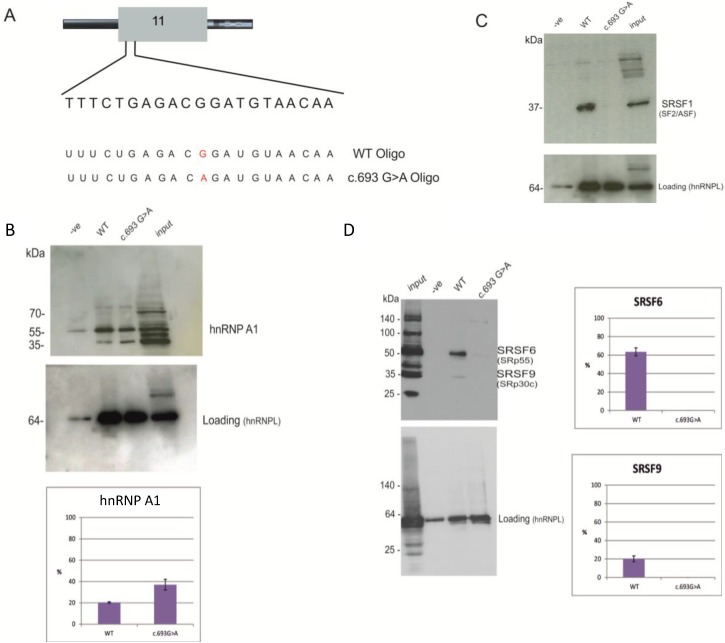
Pull down analysis of BRCA1 oligo WT and synonymous c.693G>A. (**A**) Schematic representation of the two oligonucleotides used for the pull-down assay. The WT oligo and the c.693G>A oligo carrying the variant (shown in red); (**B**) Western blot of the pull-down sample using hnRNPA1 antibody (**upper panel**) and hnRNPL (**lower panel**). hnRNPL represent the loading control. The −ve lane contain proteins purified from pull-down assays. The input lane contains 1/20 of input HeLa nuclear extract. The histograms show the % of intensity of the hnRNP A1 band calculated against the loading control using Image J. The percentages and standard deviation (error bars) are calculated from two biological replicates; (**C**) Western blot of the pull-down sample using SRSF1 antibody (**upper panel**) and hnRNPL (**lower panel**). The −ve lane contain proteins purified from pull-down assays. The input lane contains 1/20 of input HeLa nuclear extract;and (**D**) Western blot of the pull-down samples using the 1H4 antibody (**upper panel**). This antibody is able to detect several SR proteins (input lane), but only two bands corresponding to SRSF6 and SRSF9 are visible in the WT lane. hnRNPL represent the loading control (**lower panel**). The input lane contains 1/20 of input HeLa nuclear extract. The −ve lane represents the control sample from beads only, with no RNA. The WT and c.693G>A lanes contain proteins purified from pull-down assays. The histograms show the % of intensity of the SRSF6 and SRSF9 bands calculated against the loading (intensity of hnRNPL band) using Image J. The percentages and standard deviation (error bars) are calculated from two biological replicates.

To identify the trans-acting factors able to bind BRCA1 exon 11 and the variant sequence, we performed a pull-down analysis using two synthetic RNA oligonucleotides: the WT oligonucleotide and an oligonucleotide containing c.693G>A. The sequences of the RNA oligos are shown in Figure 7A. The sequence CACACACA, able to bind hnRNPL [19], was added at the 3' end of both synthetic oligonucleotides, so that data could be normalised to hnRNPL binding. Proteins binding the synthetic RNAs were analysed by sodium dodecyl sulfate polyacrylamide gel electrophoresis (SDS-PAGE) followed by Coomassie blue staining (data not shown) and western blot.

Figure 7B shows that the antibody against hnRNP A1 detects hnRNP A1 binding to the wild-type RNA oligonucleotide, as well as to the oligonucleotide with the synonymous variant, c.693C>A, although the intensity of the hnRNP A1 band relative to the loading control hnRNPL is higher in the c.693G>A lane. 

The bioinformatics approach initially suggested that the synonymous c.693G>A variant disrupted binding sites for SRSF1, SRSF5 and SRSF7; therefore, a pull-down analysis followed by western blot using antibodies to SRSF1, SRSF7 and 1H4 (which binds each member of the SR protein group) were used in order to detect binding to WT and mutant RNA oligos. 

The western blot results in Figure 7C show that SRSF1 is able to bind the wild-type RNA sequence, but not the RNA sequence with c.693G>A. In addition, the antibody, 1H4, able to detect phosphorylated SR proteins, was used. Results show that SRSF6 and SRSF9 were able to bind the RNA WT sequence only (Figure 7D). 

**Figure 8:**
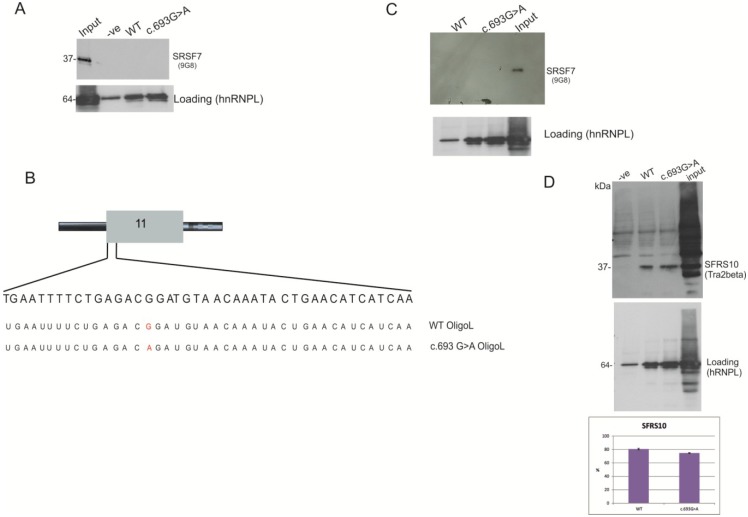
Western blot of the pull-down analysis of BRCA1 oligo WT and synonymous c.693G>A. (**A**) Western blot of the 12% polyacrylamide gel from pull-down samples using RNA short oligonucleotide WT or carrying the variation c.693G>A. the −ve lane represents a control sample from beads only with no RNA. The WT and c.693G>A lanes contain proteins purified from pull-down assays. The input lane contains 1/20 of input HeLa nuclear extract. The band detected with the hnRNPL antibody represents the loading control; (**B**) Schematic representation of the two long oligonucleotides used, the WT oligo L and the c.693G>A oligo L carrying the variant (shown in red); (**C**) Western blot from the pull-down samples using RNA long oligonucleotide WT or carrying the variation c.693G>A. The −ve lane represents a control sample from beads only with no RNA. The WT and c.693G>A lanes contain proteins purified from pull-down assays. The input lane contains 1/20 of input HeLa nuclear extract. The hnRNPL antibody represents the loading control;and (**D**) Western blot of the pull-down samples using Tra2beta antibody. hnRNPL represent the loading control. The histograms show the % of intensity of the Tra2beta band calculated against the loading (intensity of hnRNPL band) using Image J. The percentages and standard deviation (error bars) are calculated from two biological replicates.

The Human Splicing Finder predicted that in the presence of the variant, c.693G>A, the binding site for SRSF7 is disrupted. However, when using an antibody against SRSF7, we were unable to detect any binding, either in wild-type oligo or the RNA oligo, with variant c.693G>A (Figure 8). This may be because the binding of SRSF7 to RNA could require the binding of accessory SR proteins, in particular Tra2beta [20]. The Human Splicing Finder predicts binding sequences for Tra2beta down-stream of the putative binding sequence for SRSF7 (data not shown). This region is not covered by the 21 synthetic RNA oligonucleotides used for the pull-down analysis. Consequently, a different synthetic RNA oligonucleotide spanning a longer region was analysed in a further pull-down assay (Figure 8B). This long RNA was still unable to bind SRSF7 (Figure 8C). However, using a Tra2beta antibody, we were able to detect the presence of the protein in both the wild-type and the c.693G>A variant (Figure 8D).

In summary, the pull-down experiments suggest that SRSF1, SRSF6 and SRSF9 are the splicing factors binding the regulatory region identified in exon 11 and that their binding is disrupted in the presence of the variant, c.693G>A. The western blot analysis also demonstrated that hRNPA1 is able to bind both the wild type sequence and the mutant sequence with the c.693G>A variant. Finally, we see that Tra2beta is able to bind a region downstream of c.693. 

## 3. Experimental Section

### 3.1. Minigene Constructs

The pB1 minigene was obtained through a two-step PCR mutagenesis method [21], as previously described [14].

### 3.2. Cell Culture

Human breast cancer cell lines, MCF7 (ATCC number: HTB 22™), were grown in Dulbecco’s modified Eagle’s medium (DMEM) medium with 4500 mg/L glucose, pyruvate and l-glutamine supplemented with 1% penicillin/streptomycin and 10% foetal bovine serum. Cells were incubated at 37 °C in a 5% CO_2_ atmosphere.

### 3.3. Transfection

Minigene plasmids were transfected in MCF7 cell lines, using the FuGENE 6 transfection reagent from Roche. One hundred microlitres of DMEM serum-free medium containing 1.5 μg of vector DNA and 4 μg of FuGENE reagent were incubated for 15 min at room temperature before the mixture was added in a 6-cm well cell culture (70% confluent) in the presence of 10% foetal bovine serum. After 48 h the RNA was extracted and the splicing products were analysed as described previously [14]. Fifty six percent of the cells were efficiently transfected. 

### 3.4. Pull-Down Assay

One nanomole of RNA oligonucleotide (WT short 5'-UUUCUGAGACGGAUGUAACAA-3', WT long 5'-UGAAUUUUCUGAGACGGAUGUAACAA-3', Syn 5'-UUUCUGAGACAGAUGUAACAA-3', Syn long 5'-UGAAUUUUCUGAGACAGAUGUAACAA-3') were placed in a 400-μL reaction mixture containing 5 mM sodium m-periodate, incubated for 1 h in dark at room temperature. The RNA was ethanol precipitated and resuspended in 100 μL of 0.1 NaOAc, pH 5; 100 μL of adipic acid dehydrazide agarose bead 50% slurry (SIGMA), washed and resuspended in 300 µL of 0.1 NaOAc, pH 5, were added. This mix was incubated overnight at 4 °C on a rotator. The beads with the bound RNA were then pelleted and washed three times with 1 mL of 2 M NaCl and equilibrated in 1× RNA buffer (20 mM 4-(2-hydroxyethyl)-1-piperazineethanesulfonic acid (HEPES), pH 7.5, 0.2 mM Ethylenediaminetetraacetic acid (EDTA), 0.5 mM dithiothreitol, 6% glycerol, 100 mM KCl). They were incubated in 1× RNA buffer containing 600 μg of HeLa nuclear extract and 5 μg/μL of Heparin for 30 min at room temperature in a 500-μL final volume. The beads were then pelleted at 1000 rpm for 5 min and washed five times with 1 mL of 1× RNA buffer, before the addition of SDS sample buffer and loading onto a 12% SDS-PAGE gel. 

### 3.5. Western Blot

Pull-down samples were loaded on a 12% SDS-PAGE gel and electro-blotted onto a Hybond ECL membrane (GE-healthcare, Chalfont St Giles, UK), and antibody recognition was then performed using in-house antibodies against hnRNP A1 and commercial antibodies against SRSF1 (Invitrogen, Carlsbad, CA, USA), against SRSF6 and SRSF9 (1H4, Invitrogen) and against 9G8, Tra2beta and hnRNPL (Abcam, Cambridge, UK). Protein bands were detected using the ECL kit (Pierce, Rockford, IL, USA), according to manufacturer’s protocol. 

### 3.6. In Silico Predictions

The Human Splicing Finder [17,18] was the tool used to predict putative splicing regulatory sequences in BRCA1 exon 11. Two sequences were analysed for the effect of the variant c.693G>A on splicing. The wild-type sequence is as follows: 

AGCTGCTTGTGAATTTTCTGAGACGGATGTAACAAATACTGAACATCAT. 

The mutant sequence (the synonymous change c.693G>A is shown in red) is as follows: AGCTGCTTGTGAATTTTCTGAGACAGATGTAACAAATACTGAACATCAT.

The two sequences were run in the Human Splicing Finder program choosing the option “analyse mutations” from the drop down menu. 

## 4. Conclusions

We have evaluated the effect and role of the synonymous variant c.693G>A in BRCA1 exon 11 and shown how this affects the process of splicing. We show that both when using RNA from a blood sample from a patient and *in vitro* (using a minigene for the splicing assay), the sequence variant increases Δ11 and decreases the Δ11q and full 11 isoforms of the *BRCA1* gene, thus altering the balance of the BRCA1 isoforms (Figure 1 and Figure 2). Although this variant was not originally considered pathogenic [13], it is still possible that this variant leads to cancer predisposition. It is already known that BRCA1 exon 11 splicing isoforms are implicated in cancer [9,22,23], and maintaining the correct isoform proportions is important in preventing cell transformation. Exon 11 of BRCA1 is the largest exon of the gene and encodes two putative localization signals (NLS); it also contains a domain that interacts with RAD51. BRCA1 interacts with RAD51 to repair DNA damage, in the HR (homologous repair) pathways. Deletion of exon 11 could alter the mechanism of DNA repair and, therefore, alter the integrity of the genome, which can, in turn, be linked with an increment of the lifetime risk of breast and ovarian cancer. The Δ11q and Δ11 isoforms lack nuclear localization signals (NLS), but they enter the nucleus through an alternative mechanism that requires Ubc9 or via the RING-domain-mediated BARD1 (BRCA1-associated RING domain protein 1) import pathways [24,25]. Overexpression of the BRCA1 Δ11q isoform can elevate nuclear levels and, consequently, increase apoptosis. Alternatively, cytoplasmic retention of Δ11q may induce cell proliferation. 

In this study, we verified, using a minigene containing from exon 8 to exon 12 of the *BRCA1* gene, that the synonymous variant gave the same splicing outcome observed in the patient RNA, confirming that this assay is a useful tool for testing the effect of unclassified variants on splicing in these exons. This is important, as exon 11 is a large exon, where it has been notoriously difficult to assess the effect of variants on RNA. 

The minigene splicing assay is also a useful system to study the mechanistic effect of splicing. In several studies, we have been able to identify splicing regulatory sequences using the site-directed mutagenesis of positions surrounding splicing mutations in minigene systems [26,27,28].

In this study, site-directed mutagenesis using the pB1 minigene suggested overlapping enhancer and silencer functions of the region surrounding position c.693 and, therefore, the presence of a putative CERES (Figure 3 and Figure 4). 

CERESs have been previously demonstrated in CFTR (cystic fibrosis transmembrane conductance regulator) exon 9 and exon 12 [15,16,17,29]. In CFTR exon 12, two CERES regions have been identified, CERES 1 and CERES 2, which appear to be context dependent for splicing. It was shown that the CERES 2 in CFTR exon 12 could bind a number of different trans-acting factors (in particular, SRSF1, SRSF6 and hnRNP A/B), even though CERES 2 has a short RNA sequence (<10 nucleotides). The identification of CERES with the same sequences for enhancer and silencer elements have also been found in CFTR exon 9, indicating a common splicing regulator role of these elements. There are also other examples of small exons where enhancer and silencer factors (in particular, SRSF1 and hnRNP) can bind the same regions, (e.g., SMN (survival motor neuron protein) exon 7) [30,31,32].

The presence of these overlapping regulatory elements in *BRCA1* makes exon 11 an interesting and complicated exon for analysis. The putative CERES element in this case appears to span at least the whole region studied.

As well as demonstrating the presence of a composite regulatory element at the beginning of exon 11, we also undertook experiments in which a double mutant at codon positions c.689 and c.693 was incorporated into the minigene. This demonstrated that c.689A>C can restore the inclusion of exon 11 caused by synonymous substitution of c.693G>A (Figure 5) in a compensatory manner. 

We found that the variant, c.693G>A, disrupts the binding of SRSF1, SRSF6 and SRSF9 to the CERES *in vitro* (Figure 7). These proteins enhance splicing and usually bind exonic RNA sequence, as well as aid the splice site recognition of exon and intron junctions [33,34,35,36]. Our results support the hypothesis that SR proteins regulate the inclusion of exon 11. In addition, a silencer hnRNP A1 was found to bind the CERES region *in vitro*,both in the presence of the wild-type sequence and the variant change, c.693G>A, with an increase in the presence of the variant. HnRNP A1 can therefore also contribute to the silencer feature of the CERES element in BRCA1 exon 11. Interestingly, Brando *et al.* [13] have since reported a c.692G>A variant in their paper characterizing unclassified variants in the *BRCA*1 and *BRCA*2 genes.

Whilst exclusion of exon 11, due to the synonymous variant, c.693G>A, had already been reported in the literature [13], in our study, we also saw a relative decrease of the Δ11q isoform in the variant compared to wild type. Different primers and experimental conditions in a splicing assay can affect results to some degree [37], and this may explain why some of our results differ from those reported previously. We plan to assess the functional effects of this element in the future using RNAseq. While this will not differentiate cause and effect, it will give some insight into what the global changes are that follow that 693G>A transition. 

Overall, it is clear that the unclassified variant, c.693G>A, affects the splicing process of exon 11 in the *BRCA1* gene. In addition, our experiments point to the presence of a composite regulatory element, within which different SR proteins (SRSF1, SRSF6 and SRSF9) and hnRNP A1 are able to bind, *in vitro*, and whose binding is disrupted in the presence of the unclassified variant, c.693G>A. That a composite regulatory element exists at the beginning of exon 11 of the *BRCA1* gene illustrates the difficulty of identifying which splicing factors are responsible for the exclusion or inclusion of complex exons and, in particular, the BRCA1 exon 11. 
